# Challenges and strategies for improving medication adherence among adolescent psychiatric patients: A qualitative study

**DOI:** 10.1017/gmh.2024.80

**Published:** 2024-12-02

**Authors:** Clarisse Roswini Kalaman, Norhayati Ibrahim, Choy Qing Cham, Meng Chuan Ho, Uma Visvalingam, Fairuz Nazri Abdul Rahman, Farah Ahmad Shahabuddin, Mohd Radzi Tarmizi A Halim, Ching Sin Siau

**Affiliations:** 1 Center for Healthy Ageing and Wellness (H-CARE), Faculty of Health Sciences, Universiti Kebangsaan Malaysia, Kuala Lumpur, Malaysia; 2 Institute of Islam Hadhari, Universiti Kebangsaan Malaysia, Bangi, Malaysia; 3 Centre for Pre-U Studies, UCSI University (Springhill Campus), Port Dickson, Malaysia; 4 Department of Psychiatry, Hospital Putrajaya, Putrajaya, Malaysia; 5 Psychiatry Department, Faculty of Medicine, Universiti Kebangsaan Malaysia, Kuala Lumpur, Malaysia; 6 Department of Psychiatry, Hospital Bahagia Ulu Kinta, Tanjung Rambutan, Malaysia; 7 Faculty of Business, Economics and Human Development, University Malaysia Terengganu, Kuala Terengganu, Malaysia; 8 Centre for Community Health Studies (ReaCH), Faculty of Health Sciences, Universiti Kebangsaan Malaysia, Kuala Lumpur, Malaysia

**Keywords:** Challenges, medication adherence, qualitative study, adolescent psychiatric patient, Malaysia

## Abstract

Despite significant advancements in the development of psychotropic medications, increasing adherence rates remain a challenge in the treatment and management of psychiatric disorders. The purpose of this study is to qualitatively explore the challenges underlying medication adherence and strategies to improve it among adolescents with psychiatric disorders in Malaysia. This qualitative research design presents results from 17 semi-structured interviews with adolescent psychiatric patients, aged 11 to 19 years old, from public hospitals across Peninsular Malaysia. The data collected from interviews were transcribed and processed through thematic analysis using the NVivo 11 software. A total of three main themes concerning medication adherence were identified: (1) challenges; (2) coping strategies and (3) protective factors. In this study, thirteen subthemes emerge as challenges underlying medication adherence experienced by adolescent psychiatric patients. The coping strategies identified in this study fall under three broad subthemes which are problem-focused strategies, emotion-focused strategies and maladaptive strategies. This study also highlights social support and positive medicinal effects as protective factors for non-adherence issues in adolescent psychiatric patients. In conclusion, this study supports the notion that adherence is a multi-factorial phenomenon. This study can inform future development of interventions and targeted health promotion programmes in enhancing adherence.

## Impact Statement

Medication non-adherence can cause substantial worsening of mental health disorders in adolescent psychiatric patients. Therefore, this study presents a deeper dive into a better understanding of the challenges that cause non-adherence and coping strategies to enhance adherence, particularly among adolescents with psychiatric disorders in Malaysia. By identifying challenges associated with medication non-adherence, policies and tailored interventions can be developed. This study is written as a continued effort to advocate for changes to current global mental health practices and treatments among adolescents.

## Introduction

The prevalence of children and adolescents being diagnosed with psychiatric disorders, globally and in Malaysia, is significantly increasing (Institute for Public Health, 2017; World Health Organization [WHO], 2021). According to the WHO (2003), psychotropic medications are the most common form of treatment among psychiatric patients. Hence, adherence, particularly medication adherence, is an important determinant of psychiatric treatment outcomes (Novick et al., 2015). Even though considerable progress has been made in the development of psychotropic medications, improving the rates of adherence in the management of psychiatric disorders remains a worldwide problem (Neiman et al., 2017; Ramamurthy et al., 2023; Semahegn et al., 2020; WHO, 2003). Medication adherence can be defined as “the extent to which a person’s behaviour corresponds with agreed recommendations from a health care provider” (WHO, 2003, pp. 3). This includes taking the prescribed number of pills according to the prescribed dosage, time, frequency and direction (Cutler & Everett, 2010; Osterberg & Blaschke, 2005).

Non-adherence, intentional or unintentional, can be demonstrated in various forms, such as missed doses, premature discontinuation of medication, alteration of medication prescription, overdose and drug holidays (Jimmy & Jose, 2011; WHO, 2003). In the many measures of adherence, a cut-off point of 80% was used to distinguish adherence from non-adherence behaviour (Hatah et al., 2020; Morisky et al., 1986; Thompson et al., 2000). According to WHO (2003), there are no optimal estimates of adherence or non-adherence, but rather a multi-method approach (e.g., combining self-reporting and objective measures) should be employed in measuring adherence behaviours. In view of this, recent studies have reported that the prevalence of non-adherence for people with psychiatric disorders was in the range of 30%–65% whilst the prevalence of non-adherence among children and adolescents with psychiatric disorders was in the range of 6%–69% (Edgcomb & Zima, 2018; Hage et al., 2016; Kalaman et al., 2023; Ramamurthy et al., 2023; Semahegn et al., 2020; Timlin et al., 2015). Data on the adherence rates among psychiatric patients, particularly among children and adolescents, in Malaysia are limited. However, according to the Clinical Practice Guidelines for Management of Schizophrenia (Malaysian Health Technology Assessment Section [MaHTAS], 2021), non-adherence rates among people with schizophrenia were 50%–70%. Similarly, the Clinical Practice Guidelines of Management of Bipolar Disorder (MaHTAS, 2014) reported a range of 19%–69% treatment non-adherence rates among people with bipolar disorder. Therefore, improving adherence among people with psychiatric disorders, despite the age group, in the Malaysian context, is essential for achieving the maximum benefit of medication and thus promoting better treatment outcomes (MaHTAS, 2014, 2019, 2021).

There are several challenges patients reported facing in achieving medication adherence, which all contribute to the increase of non-adherence and its consequences, such as morbidity, mortality, additional medical expenses and lower quality of life (Farrukh et al., 2021; Malik et al., 2020; Neiman et al., 2017). According to the WHO (2003), patients can encounter life-threatening risks such as intense relapses, increased dependency or toxicity with medicines, drug resistance possibilities and increased likelihood of experiencing drug rebound effects. It is important to address these challenges as it represents a significant step in improving adherence, especially among psychiatric patients, who encounter additional challenges such as suicidal ideation, mood swings and public stigmatization (Goldstein et al., 2016; Levin et al., 2016). In the many studies conducted on medication adherence among patients with psychiatric disorders, reasons for non-adherence were deemed as a multifactorial phenomenon (Gebeheyu et al., 2019; Gudeta et al., 2023; Kvarnström et al., 2021; Phan, 2016; Semahegn et al., 2020; WHO, 2003). However, according to Kalaman et al., (2023), since children and adolescents are under the purview of parental caregivers, achieving adherence among children and adolescents with psychiatric disorders may require an added emphasis on parental factors. In contrast, several studies reported that adolescents, compared to children and adults, are more vulnerable to medication non-adherence (Brinkman et al., 2016; Buitelaar, 2017; WHO, 2003). Adolescents’ vulnerability to non-adherence can be due to the onset of physical and psychological development implicit in adolescence (WHO, 2003). Therefore, further studies in addressing adherence issues among adolescents should be conducted.

Given the importance of medication adherence, a deeper understanding of the challenges experienced, particularly among adolescents with a psychiatric disorder in Malaysia, will help inform interventions relevant to our context (Dikec et al., 2022; Ho et al., 2017; Zarea et al., 2016). A qualitative study design may facilitate the emergence of information on medication adherence challenges unique to the Malaysian context, such as family dynamics, cultural influences, social support and other environmental factors. A qualitative study also explores the adolescent patient’s subjective experiences and uncovers underlying reasons for non-adherence (Clifford et al., 2020; Ho et al., 2017). Therefore, this study seeks to explore the themes that emerge on challenges underlying medication adherence and strategies to improve it in adolescents with psychiatric disorders in Malaysia using a qualitative design.

## Methodology

### Study Design

An exploratory, qualitative research design was employed in this study. This study followed the Consolidated Criteria for Reporting Qualitative Research (COREQ; Tong et al., 2007).

### Participants

Purposive sampling was used to recruit adolescent psychiatric patients, aged 11 to 19 years old, who are being prescribed psychiatric medications. The inclusion criteria of participants in this study include: (1) attending an inpatient or outpatient psychiatric facility; (2) being diagnosed with a psychiatric disorder; (3) being prescribed psychiatric medications by psychiatrists; (4) being able to understand and speak Bahasa Malaysia; (5) being able to provide informed assent (if below 18 years old) or informed consent (if 18 years old and above); and (6) a parent or guardian providing informed consent for their child/ward (if below 18 years old) to participate in this study. Participants experiencing active psychosis or possessing any visual or auditory impairment that could compromise participants’ ability to understand or follow the study directions were excluded from the study. Participants were screened and recruited upon referral from professional psychiatrists. All 17 adolescent psychiatric patients who were screened were recruited for this study. The process of recruiting and interviewing participants was concluded when data saturation was achieved (Boddy, 2016; Guest et al., 2006; Mason, 2010). Data analysis was conducted in parallel with interviews, allowing researchers to reaffirm data saturation in the subsequent 2 interviews after achieving saturation with patient 15.

### Procedures

Participants were briefed on the purpose, procedures and ethical principles of the study. For those who were under 18 years old, both the adolescent and their parent/guardian were briefed. Informed consent was provided by participants who were 18 years of age and older. Adolescents below 18 years old provided informed assent, whilst their parents/guardians provided informed consent. Then, according to the participant’s availability and preference, the mode (online or face-to-face) and time of the in-depth interview were scheduled. Six of the interviews took place in private rooms at public hospitals, whilst the remaining eleven interviews were conducted online via Zoom, with only the interviewer present. Both the face-to-face and online interviews carried out in this study varied in length from 20 minutes to approximately 1 hour. The interviewees were aware of the interviewer’s background and had no prior or ongoing relationship with the interviewer. With participants’ consent, all interviews were audio recorded and transcribed verbatim into Bahasa Malaysia or English. Since this study complies with the Declaration of Helsinki, interview transcripts were anonymized (e.g., removing names of people and places) to preserve participant confidentiality. Peer debriefing strategies were utilized in this study to enhance the validity of the information gathered from the interviews (Creswell & Creswell, 2018). Transcripts were distributed to the research team and discussions on ways to improve interview techniques were conducted throughout the data collection process to ensure richer qualitative data were captured.

The interview guide developed by the research team was semi-structured. The core interview questions were developed in accordance with the aims of this study, an extensive literature review, and input from experts in the field. The interview questions were developed in Bahasa Malaysia and finalised by the multidisciplinary research team after receiving input from an external evaluator. The evaluator, who is a registered counsellor, evaluated the interview questions based on their coverage of the study aims and its applicability to the target population. The interview guide is presented in Table A1. The interview guide was pilot-tested with a non-participant adolescent psychiatric patient, which resulted in a few wording changes. Due to the semi-structured nature of interviews, apart from the core questions in the guide, probing questions were asked in areas that needed more clarification or elaboration.

### Data Analysis

The thematic analysis conducted in this study was guided by the six steps outlined by Braun and Clarke (2006, 2021). All interview transcripts were thematically analysed in the original language using the N-VIVO version 11 software (QSR International Ltd., Australia) by C.R.K. and reviewed whilst listening to the recording by C.Q.C. to ensure accuracy and consistency. Participants’ personal identifiers, as well as demographic profiles (i.e., age, race, religion), were removed from the transcripts to reduce the risk of biases in the analysis. Noticeable pauses and non-verbal behaviour such as laughter were included in the transcripts, as were interruptions and inaudible words. The interviews were transcribed as soon as possible following the occurrence and were aligned with the interviewer’s field notes to prevent any ambiguity and inaccuracies. Then the data were systematically coded and collated by C.R.K., N.I. and S.C.S. to generate meaningful themes. The coding process begins with the identification of meaningful codes within the transcripts. Codes identified were then grouped into categories and the naming of themes was conducted based on the connections made between the categories. There was consensus about the codes, categories and themes developed among the researchers.

An inter-rater reliability analysis using Cohen’s Kappa statistic was performed by three professionals (i.e., a child and adolescent psychiatrist, a senior nursing lecturer and a licenced professional counsellor) to determine the level of agreement on themes, subthemes and codes among raters. Cohen’s Kappa value of κ =.96 was obtained and was considered an almost perfect rate of agreement (Cohen, 1960; Landis & Koch, 1977). The final themes were confirmed by discussion and consultation within the research team until consensus was achieved.

## Results

### Participant characteristics

There was a total of 17 participants. As shown in Table A2, most of the participants in this study were female (*n* = 13), Malay (*n* = 13) and Muslim (*n* = 13). The mean age of participants was 16.53 years (SD = 1.91; Table A2).

All participants in this study reported non-adherence to medication. The types of medication non-adherence reported by participants in this study were either erratic non-adherence (*n* = 10) and/or intelligent non-adherence (*n* = 10). Skipping dose(s) (*n* = 3), taking medications depending on symptoms instead of prescribed timing (*n* = 5), overdosing (*n* = 1) and altering medication dosage without a doctor’s advice (*n* = 1) were among the types of intelligent non-adherence participants reported.

### Themes and Subthemes

As shown in Table A3, the findings of this study explain a range of challenges, coping strategies and protective factors for improving medication adherence among adolescent psychiatric patients. A total of 37 codes were classified into 3 main themes and 18 subthemes.

**Theme 1: Medication adherence challenges.** Participants reported various challenges with medication taking. In this regard, thirteen challenges were identified.

***Subtheme 1: Medicine side effects.*** All participants indicated that it was challenging to remain adherent to medication when side effects (physical, emotional and cognitive) were part and parcel of medication taking. Most participants (*n* = 16) reported experiencing physical side effects such as drowsiness, loss of appetite, nausea, weight gain, fever, change in menstrual period timing and indigestion:

*Like this Quetiapine, if I eat, I will feel very sleepy until I cannot do my work.*

*(P05, Female, 19)*

Several participants (*n* = 6) stated having noticed that their emotional state had changed, such as the blunting of emotions:

*… the other is like, after they increase the dosage, it cancelled out all the other emotions, not just the sad emotions. I start to feel less sensitive towards jokes and all.*

*(P09, Female, 18)*

One participant highlighted that medication has caused significant cognitive changes, particularly in the ability to understand learning materials at school:

*I’m like slow to understand in my studies, that’s all I notice. The rest is like okay, lah. When I didn’t eat it before, it was easy for me to understand, but when I started taking this medicine, I find it difficult. I have to read it many times to understand.*

*(P02, Female, 15)*

Some participants (*n* = 3) reported non-adherence when the medication administered did not lead to significant changes in existing symptoms:

*Because for me at first, when I ate it, I felt like the medicine didn’t give me any effect. Meaning my problem or symptoms didn’t change.*

*(P12, Female, 14)*

***Subtheme 2: Forgetfulness.*** Several participants (*n* = 11) reported non-adherence due to forgetfulness:

*There are few times I legitly forget. I completely forgot where I put my medicine and the time to take it.*

*(P01, Female, 19)*

***Subtheme 3: Too busy or tired.*** Two participants stated that being busy or tired had contributed to non-adherence:

*Maybe because it was at night, I returned home from school, had a lot of activities in school, is tired, then when I reached home I just slept straight away.*

*(P15, Male, 14)*

***Subtheme 4: Procrastination behaviour.*** Participants (*n* = 2) were more likely to inadvertently skip a dose(s) when medication taking is delayed or postponed:

*That morning, I will remind myself that I have to take the medicines later at night, but when the night comes, I will still remember to take them but, I’ll be like later lah. But then I will unintentionally fall asleep.*

*(P11, Female, 14)*

***Subtheme 5: Non-adherent behaviour.*** Some participants (*n* = 3) in this study portrayed intentional non-adherent behaviours:

*I did purposely didn’t take my medicine. I purposely didn’t eat it because I didn’t think it was necessary before this. But recently, I am taking my medicine.*

*(P13, Female, 16)*

***Subtheme 6: Lack of knowledge or insights about the importance of taking medication and medication adherence.*** Participants’ (*n* = 7) lack of knowledge or insights, specifically on the importance of medication adherence, were reported to influence adherence behaviour:

*To be exact, I am not sure if taking medication at the exact time is important. I think it depends la. I just take it when I can.*

*(P16, Female, 17)*

*I don’t have any medication background. So, for me, it was like, “what this medicine is going to do”? … I think it depends on the severity that we take medicine because medication may help you. So, I think it’s compulsory to take medicine according to the instructions at the beginning. Then, if your symptoms are not severe, you can maybe take it when needed la.*

*(P06, Male, 19)*

***Subtheme 7: Negative thinking towards medication.*** Participant’s personal thoughts, such as fear of medication dependence (*n* = 4), fear that medicine will harm health in the future (*n* = 3) and feelings of dislike towards medication (*n* = 2), contributed negatively towards medication adherence:

*Sometimes I also read that this medicine cannot be taken from a younger age, I read on the internet that it is not good because it can damage the kidneys … I am worried that my life will be dependent on the medicine.*

*(P10, Female, 16)*

*I think I changed my medications 3 times because I am the kind that doesn’t like to take medicine, so every time I will skip or just choose to not eat it at all.*

*(P12, Female, 14)*

***Subtheme 8: Stigma regarding psychiatric medication.*** Participants expressed concerns about stigma regarding psychiatric medication from family and significant others (*n* = 9) as well as perceived stigma, which possibly originated from cultural influences, mass media, or society (*n* = 14). One of the participants noted that the stigma surrounding psychiatric medications had instilled a great fear about medication outcomes:

*I heard one of my friends say, if I start taking medication and if one day, I didn’t take it, they say I will be far worse and might be called crazy. So, when the doctor said I need to take medicine, I felt so scared cause I am like, “If I take it, I might get worse than ever”.*

*(P01, Female, 19)*

Another participant reported the possibility of experiencing a sense of distress if her condition was disclosed to others:

*No. I keep it a secret. I just be quiet lah. I think that they will say like “Why you need pill? Are you crazy? You don’t need pill and all.” If I hear those words, I will feel like “You don’t know my situation”, so I will also feel down lah.*

*(P14, Female, 14)*

***Subtheme 9: Negative feelings towards medication.*** Some of the negative feelings reported by participants in this study were denial (*n* = 3), annoyance (*n* = 1), ignorance (*n* = 3), shame (*n* = 3) and depressed mood (*n* = 1). Negative feelings towards medication are prompted by stigma about medication, medication regimes and/or other personal experiences with medication, which were commonly reported as a significant barrier to adherence and a barrier that participants must overcome in the medication-taking process:

*Early on at first, I was like, my first reaction was like “Oh is there something wrong with me” and then from there I kept telling myself, that “I am normal, then why do I need to take medicine.” So at first, I can say that many medicines were wasted.*

*(P17, Female, 18)*

*No, but I don’t like taking medicine. So I was like “Medicine! Medicine again!” and want to skip or not eat it at all.*

*(P12, Female, 14)*

***Subtheme 10: The COVID-19 Situation.*** The COVID-19 pandemic, which led to the movement control order in Malaysia, was seen as a challenge in the medication-taking process by two participants in this study. Participants were not able to restock their medications on time due to movement restrictions. This situation led participants to think that since the virus was very deadly, mental health care was not a priority at the time.

***Subtheme 11: Familial factors.*** Participants (*n* = 10) described negative family relationships (*n* = 5) and a lack of family involvement (*n* = 4) as well as support (*n* = 4) was associated with poorer adherence. One participant described dysfunctional family relationships and the lack of support received as leading to intelligent non-adherence:

*I started going, I’m not sure, but I’ve been following up under this psychiatrist for 3 years, since Form 1 and now I’m Form 3. Then I go, firstly because of family problems, and until now it is still not solved. In the past, my parents and grandmother did not support me taking medicine. They said that this medicine is not good and will cause quite bad effects that will come later. I was forced to follow what they said.*

*(P05, Female, 15)*

whilst another participant described how the lack of family involvement in the medication-taking process has led to poorer adherence due to her inability to independently keep up with the medication regime:

*I think no because I can handle it on my own. I do not rely on anyone because I know everyone if busy. So, I have to rely on myself, have to remind myself, because my mother is busy with her work. If I remember, then I will take my medicine.*

*(P13, Female, 16)*

***Subtheme 12: Social factors.*** Another external factor that was reported to be a significant challenge in the medication-taking process was poor social relations (*n* = 7), and a lack of social support (*n* = 9). Most participants (*n* = 14) agreed that social groups can greatly impact participants’ feelings, which is in relation to participants’ medication-taking behaviour.

*I personally ran some campaigns, partnered up with media and Instagram pages, and whatnot, trying to spread awareness. But I did get backlash from that, with people saying that I am attention-seeking. That hurt me. Now, I am totally closed.*

*(P09, Female, 18)*

*I think not. They don’t really concern much. Last time when I was working and, at times, was clumsy or not able to do my job well, some colleagues looked down on me and despised me lah.*

*(P07, Male, 19)*

***Subtheme 13: Health care system factors.*** Several participants reported challenging issues faced in the Child and Adolescent Mental Health Services (CAMHS) system (*n* = 3):

*During the beginning, when I started to take pills, my psychiatrists reminded me to take pills, which is something you don’t get in a general hospital. The most important part is that when I need an immediate session, like when I have suicidal thoughts, I can schedule a session with my psychiatrists the next day itself. But in government hospitals, you have to go through an entire process, and there is like a waiting list.*

*(P09, Female, 18)*

Some participants reported dissatisfaction with psychiatrists (*n* = 3) as a challenge faced in the medication-taking process:

*Urmm‥ I want to know what my medicine is like. I asked the doctor but the doctor couldn’t explain it to me.*

*(P05, Female, 15)*

**Theme 2: Medication Adherence Coping Strategies.** A total of ten coping strategies, grouped into three main themes, were observed in this study.

***Subtheme 1: Emotion-Focused Strategies.*** The most common emotion-focused strategy participants (*n* = 9) used in dealing with medication adherence challenges was accepting that they were ill:

*I feel we should just accept la. Accept that you have this disease, then you must take medicine. You just follow their instructions, just do it and see lah.*

*(P08, Male, 18)*

Some participants positively reframed thoughts (*n* = 8) and were medically adherent in order to achieve their personal goals:

*I am studying Psychology in x College. Because I have these intrusive thoughts, so I was interested in this course and want to study this course also. So, I have to cure myself before I cure someone else.*

*(P08, Male, 18)*

One of the participants used humour to reduce the perceived stress of taking psychiatric medication:

*I really don’t mind. Even me and my family will make jokes about it, like “ahh ahh, the whole family have to take medicine, the whole family is crazy”.*

*(P03, Female, 17)*

***Subtheme 2: Problem Focused Strategies.*** All participants in this study reported applying problem-focused strategies, such as active coping (*n* = 9), gaining social support from family, friends and psychiatrists (*n* = 16), as well as utilising instrumental support (*n* = 6), in the effort to stay medically adherent:

*So far gaining weight is the major problem. I just have to be careful with what I eat and I try not to overeat lor.*

*(P07, Male, 19)*

*Yes. They do know. I ask my best friend, she even remind me because we always eat lunch together in school, and whenever we are eating she will be like “weh your medicine”. I also have a reminder on my phone to remember taking medicine.*

*(P03, Female, 17)*

***Subtheme 3: Maladaptive Coping Strategies.*** Some participants relied on maladaptive coping strategies, such as the use of food and beverages (*n* = 1), behavioural disengagement (*n* = 4), self-distraction (*n* = 1) and self-blame (*n* = 1), to cope with medication side effects:

*I remember drinking a lot of coffee and Redbull to cope with the side effects. I mean like 3 cups of coffee managed to keep me awake.*

*(P09, Female, 18)*

**Theme 3: Medication adherence protective factors.** Amidst the challenges and strategies in improving medication adherence described by participants, two significant protective factors were identified in this study.

***Subtheme 1: Social support.*** Most participants (*n* = 11) agreed that strong social support from family members, friends, teachers and psychiatrists provided encouragement for participants to maintain adherence:

*Most of my family members know. They did ask a bit more but after that they were okay with it. Surprisingly, they were quite supportive. It was also okay sharing with my friends because they understood. They said I was brave enough to go to the psychiatrists and encouraged me.*

*(P04, Female, 18)*

*Alhamdulilah. My class teacher is positive. He/She supported me to continue striving.*

*(P15, Male, 14)*

*No. Even though mine was government hospital, the staffs was very welcoming. They were very open about everything, so it was super convenient. And they make me feel comfortable, that’s the most important thing during this journey.*

*(P06, Male, 19)*

***Subtheme 2: Feeling better because of the medicine.*** When asked about the reason participants still adhere to medications despite the challenges faced, almost all participants (*n* = 16) reflected on past positive experiences with medications. Participants recalled feeling better and experienced symptom relief after medications:

*I think it’s helpful for me and it helped me a lot, because I know that this is like some stigma, you know going to the mental hospital and taking pills, there is a taboo but I think going there has helped me a bunch.*

*(P03, Female, 17)*

## Discussion

Findings from the present qualitative study explored the challenges underlying medication adherence and coping strategies employed to improve adherence among adolescents with psychiatric disorders in Malaysia. The main findings of this study suggest that patient-related challenges (i.e., medicinal side effects, intentional or unintentional behaviours, stigma, lack of knowledge and insights), social challenges (i.e., dysfunctional relationships, stigma or discriminatory conduct, lack of involvement), health care team/system-related challenges (i.e., therapeutic alliances, shortage of manpower and medication stock) and pandemic-related factors contribute to the reasons of non-adherence among adolescent psychiatric patients in Malaysia. Adolescents engaged in both adaptive and maladaptive coping strategies to deal with non-adherence. In addition, the presence of social support and perceived positive medicinal side effects were identified as protective factors in this study.

A prominent challenge experienced by participants was the side effects of medications. According to Emilsson et al. (2016), adolescents with fewer perceived side effects tended to be more medically adherent. Likewise, adolescents in this study reported the struggles of adhering to medication due to its unpleasant physical, emotional and cognitive side effects. Additionally, the absence of the desired effect of alleviating psychiatric symptoms led the adolescents to believe that the medication prescribed was not beneficial. Consistent with past studies, a sense of uncertainty or distrust in the efficacy of medications can negatively affect adherence levels in adolescents (Dikec et al., 2022; Gray & Deane, 2016; Marrero et al., 2020; McMillan et al., 2020).

This study showed that adolescents used both adaptive and maladaptive coping strategies to deal with non-adherence challenges. Aside from active coping strategies such as rescheduling and implementing healthy habits, adolescents also tend to resolve to maladaptive coping strategies such as the use of caffeinated drinks and other medications, behavioural disengagement and self-distractions. According to a qualitative study conducted by Blixen et al. (2016) on coping strategies used by poorly adherent patients with bipolar disorder, maladaptive coping strategies that were identified were replaced with better coping strategies (e.g., problem-focused and emotion-focused strategies). Maladaptive coping strategies pose negative consequences to adolescent’s physical health and will likely aggravate existing mental health conditions (Addy et al., 2021; Murphy et al., 2015). Corresponding to previous studies, despite the unpleasant side effects adolescents experienced, almost all adolescents in this study identified and expressed the positive effects of medications, such as symptom reduction, improvement in social skills, increased self-confidence and improved attention in tasks, as a protective factor against medication non-adherence (Clifford et al., 2020; Dikec et al., 2022).

Patient-related challenges in taking medicine were overwhelmingly the main reasons for unintentional and intentional medication non-adherence among adolescents with psychiatric disorders. Several studies have reported unintentional behaviours, such as forgetfulness, which may not only be the reason for non-adherence but also the cause of overdose (Dikec et al., 2022; Stentzel et al., 2018). According to Elhosary et al. (2023), forgetfulness can be worsened by a lack of supervision by parents, especially among adolescents with cognitive difficulties. In contrast to findings from McMillan et al. (2020), which stated the loss of adolescents’ autonomy when parents intervene in medication regimes, the adolescents in this study reported actively relying on prompts from family and friends in coping with forgetfulness and ‘being busy or tired’ to take medicine. Similar to other past studies, the importance of phone applications, alarms and reminder notes in dealing with adherence challenges was also highlighted by adolescents in this study (Clifford et al., 2020; Dikec et al., 2022; Ho et al., 2017; Stentzel et al., 2018).

Intentional behaviours such as procrastination, non-adherence on purpose, adolescents’ negative thinking and feelings towards medication, as well as perceived stigma, were major patient-related challenges faced by adolescents in this study. Given the unique physical and psychological changes that are implicit in adolescence, this study’s findings regarding intentional non-adherence and adolescents’ negative feelings towards medication as adherence challenges are consistent with previous studies (Dikec et al., 2022; McMillan et al., 2020; Osterberg & Blaschke, 2005). Like previous studies, adolescents expressed a sense of acceptance and the use of humour in coping with negative feelings towards medication (Levin et al., 2016; Timlin et al., 2015; Zarea et al., 2016). Unlike findings from a study conducted by Ho et al. (2017), adolescents in this study reported that the perceived stigma of psychiatric medications as well as negative thinking, such as fear of medication dependence, medication dislikes and fear that medicine will harm health in the future, were not formed from religious or cultural beliefs but rather false beliefs obtained from society. Several studies have reported that Eastern cultures tend to hold more negative beliefs and stigmatization of psychiatric medications as compared to Westerners (Deng et al., 2022; Ho et al., 2017; Zarea et al., 2016). Yet the findings of this study attest otherwise. A study conducted by Gudeta et al. (2023) found that perceived stigma, when reframed differently and accompanied by a stronger belief in the necessity to medicate, can be a contributing factor in increasing adherence. Similarly, coping strategies such as positive reframing of stigmatising statements, have enabled adolescents in this study to stay adherent. This study’s findings could argue that adolescents’ limited knowledge and impaired insights regarding the effects of medication in minimising symptom severity, could be the underlying cause of stigma and non-adherence (Dikec et al., 2022; Levin et al., 2016; McMillan et al., 2020; Tessier et al., 2017; Zarea et al., 2016). This finding supports the need for psychoeducational interventions among adolescents with psychiatric disorders.

Stigma, experienced by family, friends and significant others, is one of the common social-level challenges faced by adolescents in this study (McMillan et al., 2020). When asked to further elaborate on stigmatising statements made by family and friends, adolescents in this study expressed not receiving accurate explanations. This can be attributed to the inadequate knowledge and scarcity of resources possessed by family and friends regarding psychiatric medications (Deng et al., 2022; Marrero et al., 2020; Zarea et al., 2016). According to a qualitative study conducted by Hanafiah and Bortel (2015), the prevalence of stigma surrounding mental illness in Malaysia is high, and family and friends were reported as the main perpetrators of discriminatory conduct. Consistent with previous findings, social stigma has constrained proper adherence and is a significant challenge in improving adolescents’ mental well-being (Chai et al., 2021; Deng et al., 2022; Dikec et al., 2022; McMillan et al., 2020). Family and friends do play a major role in helping adolescents with psychiatric disorders cope with medication adherence challenges (WHO, 2003). As aforementioned, even if adolescents are capable of sole responsibility for treatment regimens, the lack of involvement or support from families is a major challenge for adolescents who are still under the purview of a parent or parental caregiver (Kalaman et al., 2023; Marrero et al., 2020; McMillan et al., 2020; Nagae et al., 2015). In line with previous findings, adolescents in this study also reported the negative effects of conflicts and dysfunctional relationships with family members and friends on adherence (Elhosary et al., 2023; Timlin et al., 2015). Support obtained from families, social groups and health care professionals not only assisted adolescents in dealing with challenges but also served as protective factors that mitigated the adverse effects of other medication adherence challenges.

Health care team/system-related challenges were the least reported challenge of adherence reported by adolescents in this study. Therapeutic alliances, either collaborative or authoritative, were the most prominent service-related factor of adherence (Andrade-González et al., 2020; Chakrabarti, 2018). Adolescents in this study reported that beliefs and perceptions towards psychiatric medication can be shaped by health care professionals’ speech and conduct, which thereby determine medication adherence. Contrary to findings from Clifford et al. (2020), adolescents in this study were not concerned with the nature of the provider-patient relationship (i.e., collaborative, authoritative), instead, adolescents paid attention to the manner of service provided. There are several past studies that support the findings of this study on the important aspects of service provided by healthcare professionals (Ho et al., 2017; McMillan et al., 2020; Robards et al., 2018). The aspects are the display of a desire to promote patient health, active patient engagement and a non-judgmental environment. Besides that, similar to a study conducted by Deng et al. (2022), some adolescents in this study revealed that the shortcomings of the CAMHS system, such as insufficient workforce, which led to the frequent change of psychiatrists, as well as the shortage of medication stock, were part of the health care team/system-related challenges that have led to non-adherence, especially during the COVID-19 outbreak.

In contrast to WHO’s multifactorial phenomenon that highlights socioeconomic-related factors towards adherence, the present qualitative study did not identify any connection between factors pertaining to adolescents’ demographic or socioeconomic profile and medication adherence (WHO, 2003). This study supports the finding from Both et al. (2021), which stated that demographic and socioeconomic factors were considered irrelevant in predicting psychiatric adolescents’ adherence levels. However, findings from a recent study conducted by Demir et al. (2021) correspond with the findings of this qualitative study on the novelty of pandemic-related factors towards medication adherence. The effects of the movement control order due to the COVID-19 outbreak and adolescents’ focus on recovery from the COVID-19 infection made it harder for patients to obtain medications, thus resulting in impaired medication adherence. Adolescents did not use any coping strategies to resolve medication adherence challenges that were pandemic-related. According to Wang et al. (2022), pandemic-related factors are complicated and diverse, thereby limiting opportunities for adolescents to cope with it independently.

## Strengths and Limitations

Since the study findings are solely based on adolescents’ opinions and experiences, the limitations of this qualitative study include the existence of self-reporting biases. Future studies should consider triangulating findings from children and adolescents with more comprehensive perspectives (e.g., parents, teachers, healthcare professionals) to reduce biases. Moreover, the study findings cannot be generalised to adolescents beyond this study setting, as most of the adolescents in this study were female and of Malay ethnicity.

Despite the small sample size recruited in this study, data saturation did occur. Hence, findings regarding challenges and coping strategies for adherence can be applied to all populations. This qualitative study provided an in-depth understanding and contextual insight into the reasons for adolescents’ adherence and non-adherence, which is relatively sufficient to inform the development of policies and tailored interventions.

## Study Implications

The findings of this study have suggested several implications aimed at improving medication adherence among adolescents with psychiatric disorders. Given the significant impact of medication side effects and the lack of knowledge and insight among adolescents, there is a critical need for educational initiatives to inform patients about the benefits and potential side effects of medications. These initiatives can also be integrated into school curricula or community health promotion programs, to reduce stigma, improve public understanding as well as foster a supportive and non-judgmental environment that encourages adherence. This study also recognizes the importance of therapeutic alliances between healthcare providers and adolescents, especially in reviewing treatment plans or helpful strategies for improving medication adherence, such as weighing possible benefits of the medications versus possible risks of non-compliance, specifically symptom exacerbation and relapse. Policies aimed at addressing healthcare system deficiencies and improving the quality of mental health services are necessary to ensure continuity of care that could significantly improve medication adherence rates. The identification of diverse medication adherence coping strategies, such as automated reminders about dosing schedules, suggests future researchers explore the role of technology in offering innovative solutions to adherence challenges identified.

## Conclusions

In summary, although this study did not find a significant connection between adolescents’ demographic or socioeconomic profile and medication adherence, it still supports the notion that adherence is influenced by a variety of factors (e.g., social factors, health care team/system factors, therapy-related factors and patient-related factors), which aligns with the World Health Organization’s multifactorial approach model to adherence. This qualitative study has also shed light on the effects of Brief COPE strategies in response to medication adherence challenges, especially in a culturally diverse country like Malaysia. By addressing the challenges of adherence through the application of the practical coping strategies highlighted in this study, adherence levels among adolescents with psychiatric disorders can be improved.

## Data Availability

The data that support the findings of this study are available from the corresponding author, N.I., upon reasonable request.

## A. Appendices

**Table A1.** Semi-structured interview guide

Topic	Questions
Introductory questions	What medicines are you currently taking? How do you take these medicines? Can you describe it? How many times a day do you take these medicines? When did you start taking these medicines? Besides psychiatric medications, do you also take any other medications? Please name them.
Challenges faced in taking medications	Who explained to you the ways of taking these medicines? Do you think that you fully understood their explanation? Please elaborate on what helped you to/hindered you from understanding their explanation. How do you feel before taking these medicines? What are the physical/ emotional/ mental side effects that you experience after taking these medicines? Can you elaborate? Besides having to deal with side effects, are there any other difficulties that you faced in taking your medicines? If yes, can you elaborate? Besides medications from the hospital, do you also take any other external or traditional medications/ supplements? If yes, can you describe them? Have you ever taken your medicine in front of your friends or in public? Why or why not? May I know how would you feel if your friends or anyone else besides your family got to know about you having to take these medicines? Can you tell me what your family/ friends/ medical staff/ neighbours did when you were dealing with these side effects? In view of the COVID–19 pandemic, what are some of the other challenges that you face in taking medications?
Coping strategies used in dealing with medication adherence challenges	May I know how you feel towards the challenges that you experienced when taking the medicines? What will you do to manage these challenges? In your opinion, do you need anyone to assist you in taking the medicine? Why or why not? Do you think you can seek help from your family/ friends/ medical staff/ neighbours? Why or why not? In your own opinion, what do you think your family/ friends/ medical staff/ neighbours can do to help you in taking these medications?
Perceptions and views about the importance of medication adherence	In your opinion, do you know why do you take these medications? Can you tell us more? Do you take your medicine according to the time and instructions? Please share the details with us. In your own opinion, is it important to take your medicine at the right time and way? Why or why not? Do you have any friends/ family that also take medicines? If yes, can you tell us more about them? Have you attended any programmes/watched any videos/gained other knowledge on the importance of taking medicine? If yes, can you tell us more? Are you worried when taking these medicines? If yes, what were your worries? What do you hope for when taking these medicines? Do you feel you can fully recover from this disorder through the medicines? Why or why not? Are you worried that people may look down on you in the future? Why or why not?

**Table A2.** Characteristics of adolescent psychiatric patients (*n* = 17)

Demographic information	Mean (SD)	*n* (%)
Age	16.53 (1.91)	
14		4 (23.5)
15		2 (11.8)
16		2 (11.8)
17		2 (11.8)
18		4 (23.5)
19		3 (17.6)
Gender		
Male		4 (23.5)
Female		13 (76.5)
Race		
Malay		13 (76.5)
Chinese		2 (11.8)
Indian		2 (11.8)
Religion		
Islam		13 (76.5)
Buddhist		1 (5.9)
Christian		3 (17.6)

**Table A3.** Emerged themes, subthemes and codes of the study

Theme	Subtheme	Codes	References, *n* (%)
Theme 1: medication adherence challenges	Medicine side effects	Emotional	6 (23.1)
		Physical	16 (61.5)
		Cognitive	1 (3.9)
		No effect towards existing symptoms	3 (11.5)
	Forgetfulness	-	6 (100.0)
	Procrastination behaviour	-	2 (100.0)
	Too busy or tired to take medicine	-	2 (100.0)
	Non-adherent behaviour	-	3 (100.0)
	Lack of knowledge about the importance of taking medication and medication adherence	-	7 (100.0)
	Negative thinking towards medication	Fear of medication dependence	4 (44.4)
		Fear that medicine will harm health in the future	3 (33.3)
		Dislike medication	2 (22.2)
	Stigma regarding psychiatric medication	Adolescent’s experience with family members and significant others	9 (39.1)
		Adolescents’ perception	14 (60.9)
	Adolescent’s negative feelings	Denial	3 (27.3)
		Annoyed	1 (9.1)
		Ignorance	3 (27.3)
		Ashamed	3 (27.3)
		Depression	1 (9.1)
	COVID–19 situation	Infected with COVID–19	1 (50.0)
		Movement Control Order	1 (50.0)
	Familial factors	Negative family relationships	5 (38.4)
		Lack of family involvement	4 (30.8)
		Lack of family support	4 (30.8)
	Social factors	Poor social relations	7 (43.75)
		Lack of social support	9 (56.25)
	Healthcare system factors	Child and Adolescent Mental Health Services system shortcomings	3 (50.0)
		Patient dissatisfaction with psychiatrists	3 (50.0)
Theme 2: medication adherence coping strategies	Emotion-focused strategies	Positive reframing	8 (44.4)
		Acceptance	9 (50.0)
		Humour	1 (5.6)
	Problem-focused strategies	Active coping	9 (29.0)
		Social support from family, friends and psychiatrists	16 (51.6)
		Instrumental support	6 (19.4)
	Maladaptive coping strategies	Use of food and beverages	1 (14.3)
		Behavioural disengagement	4 (57.1)
		Self-distraction	1 (14.3)
		Self-blame	1 (14.3)
Theme 3: medication adherence protective factors	Social support	—	11 (100.0)
	Feeling better because of the medicine	—	16 (100.0)

## References

[r1] Addy ND, Agbozo F, Runge-Ranzinger S and Grys P (2021) Mental health difficulties, coping mechanisms and support systems among school-going adolescents in Ghana: A mixed-methods study. PLoS ONE 16(4), e0250424. 10.1371/journal.pone.0250424 33886671 PMC8062044

[r2] Andrade-González N, Hernández-Gómez A, Álvarez-Sesmero S, Gutiérrez-Rojas L, Vieta E, Reinares M and Lahera G (2020) The influence of the working alliance on the treatment and outcomes of patients with bipolar disorder: A systematic review. Journal of Affective Disorder 260, 263–271. 10.1016/j.jad.2019.09.014 31521862

[r3] Blixen C, Levin JB, Cassidy KA, Perzynski AT and Sajatovic M (2016) Coping strategies used by poorly adherent patients for self-managing bipolar disorder. Patient Preference and Adherence, 1327–1335. 10.2147/PPA.S110199. 27524888 PMC4966652

[r4] Boddy CR (2016) Sample size for qualitative research. Qualitative Market Research: An International Journal 19(4), 426–432. 10.1108/QMR-06-2016-0053

[r5] Both C, Mechler K, Niemeyer L, Jennen-Steinmetz C, Hohmann S, Schumm L, Dittman RW and Hage A (2021) Medication adherence in adolescents with psychiatric disorders. Zeitschrift für Kinder-und Jugendpsychiatrie und Psychotherapie 49(4), 295–306. 10.1024/1422-4917/a000813 34240621

[r6] Braun V and Clarke V (2006) Using thematic analysis in psychology. Qualitative Research in Psychology 3(2), 77–101. 10.1191/1478088706qp063oa

[r7] Braun V and Clarke V (2021) One size fits all? What counts as quality practice in (reflexive) thematic analysis?. Qualitative Research in Psychology 18(3), 328–352. 10.1080/14780887.2020.1769238

[r8] Brinkman WB, Baum R, Kelleher KJ, Peugh J, Gardner W, Lichtenstein P, Langberg J and Epstein JN (2016) Relationship between attention-deficit/hyperactivity disorder care and medication continuity. Journal of the American Academy of Child and Adolescent Psychiatry 55(4), 289–294. 10.1016/j.jaac.2016.02.001 27015719 PMC4808569

[r9] Buitelaar JK (2017) Optimising treatment strategies for ADHD in adolescence to minimise “lost in transition” to adulthood. Epidemiology and Psychiatric Sciences 26(5), 448–452. 10.1017/S2045796017000154 28413998 PMC6998898

[r10] Chai X, Liu Y, Mao Z and Li S (2021) Barriers to medication adherence for rural patients with mental disorders in eastern China: A qualitative study. BMC Psychiatry 21(1), 1–8. 10.1186/s12888-021-03144-y 33685432 PMC7941940

[r11] Chakrabarti S (2018) Treatment alliance and adherence in bipolar disorder. World Journal of Psychiatry 8(5), 114–124. 10.5498/wjp.v8.i5.114. 30425942 PMC6230924

[r12] Clifford L, Crabb S, Turnbull D, Hahn L and Galletly C (2020) A qualitative study of medication adherence amongst people with schizophrenia. Archives of Psychiatric Nursing 34(4), 194–199. 10.1016/j.apnu.2020.06.002 32828348

[r13] Cohen J (1960) A coefficient of agreement for nominal scales. Educational and Psychological Measurement 20(1), 37–46. 10.1177/001316446002000104

[r14] Creswell JW and Creswell JD (2018) Research Design: Qualitative, Quantitative, and Mixed Methods Approaches. Thousand Oaks: SAGE.

[r15] Cutler DM and Everett W (2010) Thinking outside the pillbox- medication adherence as a priority for health care reform. New England Journal of Medicine 362(17), 1553–1555. 10.1056/NEJMp1002305 20375400

[r16] Demir B, Guneysu E, Sancaktar M, Sahin SK, Elboga G and Altindag A (2021) Effect of the COVID-19 pandemic on medication adherence in psychiatric disorders. Medicine Science International Medical Journal 10(3), 720–724. 10.5455/medscience.2021.02.049

[r17] Deng M, Zhai S, Ouyang X, Liu Z and Ross B (2022) Factors infuencing medication adherence among patients with severe mental disorders from the perspective of mental health professionals. BMC Psychiatry 22(1), 1–11. 10.1186/s12888-021-03681-6 34996394 PMC8740063

[r18] Dikec G, Kardelen C, Pilz González L, Mohammadzadeh M, Bilaç Ö and Stock C (2022) Perceptions and experiences of adolescents with mental disorders and their parents about psychotropic medications in Turkey: A qualitative study. International Journal of Environmental Research and Public Health 19(15), 1–17. 10.3390/ijerph19159589 PMC9368300 35954954

[r19] Edgcomb JB and Zima B (2018) Medication adherence among children and adolescents with severe mental illness: A systematic review and meta-analysis. Journal of Child and Adolescent Psychopharmacology 28(8), 508–520. 10.1089/cap.2018.0040 30040434

[r20] Elhosary MY, Merranko JA, Goldstein TR, Hafeman DM, Goldstein BR, Gill MK, Hower H, Axelson, DA, Hunt JI, Yen S, Diler RS, Ryan ND, Keller MB, Weinstock LM, Strober M and Birmaher B (2023) Examining factors associated with medication adherence in youth with bipolar disorder. JAACAP Open 1(2), 105–115. 10.1016/j.jaacop.2023.06.001 39381188 PMC11460791

[r21] Emilsson M, Gustafsson PA, Öhnström G and Marteinsdottir I (2016) Beliefs regarding medication and side effects influence treatment adherence in adolescents with attention deficit hyperactivity disorder. European Child and Adolescent Psychiatry 26, 559–571. 10.1007/s00787-016-0919-1 27848023 PMC5394130

[r22] Farrukh MJ, Bakry MM, Hatah E and Jan TH (2021) Medication adherence status among patients with neurological conditions and its association with quality of life. Saudi Pharmaceutical Journal 29, 427–433. 10.1016/j.jsps.2021.04.003 34135668 PMC8180465

[r23] Gebeyehu DA, Mulat H, Bekana L, Asemamaw NT, Birarra MK, Takele WW and Angaw DA (2019) Psychotropic medication non-adherence among patients with severe mental disorder attending at Bahir Dar Felege Hiwote Referral hospital, north-west Ethiopia. BMC Research Notes 12(1), 1–6. 10.1186/s13104-019-4126-2 30808408 PMC6390330

[r24] Goldstein TR, Krantz M, Merranko J, Garcia M, Sobel L, Rodriguez C, Douaihy A, Axelson D and Birmaher B (2016) Medication Adherence among adolescents with bipolar disorder. Journal of Child and Adolescent Psychopharmacology 26(10), 864–872. 10.1089/cap.2016.0030 27419273 PMC5178003

[r25] Gray R and Deane K (2016) What is it like to take antipsychotic medication? A qualitative study of patients with first-episode psychosis. Journal of Psychiatric and Mental Health Nursing 23(2), 108–115. 10.1111/jpm.12288 26914866

[r26] Gudeta DB, Leta K, Alemu B and Kandula UR (2023) Medication adherence and associated factors among psychiatry patients at Asella Referral and Teaching Hospital in Oromia, Ethiopia: Institution based cross sectional study. PLOS ONE 18(4), e0283829. 10.1371/journal.pone.0283829 37053165 PMC10101398

[r27] Guest G, Bunce A and Johnson L (2006) How many interviews are enough? An experiment with data saturation and variability. Field Methods 18(1), 59–82. 10.1177/1525822X052799

[r28] Häge A, Weymann L, Bliznak L, Märker V, Mechler K and Dittmann RW (2016) Non adherence to psychotropic medication among adolescents- A systematic review of the literature. Zeitschrift für Kinder-und Jugendpsychiatrie und Psychotherapie 46(1), 69–78. 10.1024/1422-4917/a000505 27925499

[r29] Hanafiah AN and Van Bortel T (2015) A qualitative exploration of the perspectives of mental health professionals on stigma and discrimination of mental illness in Malaysia. International Journal of Mental Health Systems 9(1), 1*-*12. 10.1186/s13033-015-0002-1 25774215 PMC4359579

[r30] Hatah E, Rahim N, Makmor-Bakry M, Mohamed Shah N, Mohamad N, Ahmad M, Haron NH, Hwe CS, Wah ATM, Hassan F, Rahmat SS, Robert SA and Abdullah N (2020) Development and validation of Malaysia medication adherence assessment tool (MyMAAT) for diabetic patients. PLoS One 15(11), 1–17. 10.1371/journal.pone.0241909 PMC7647074 33157549

[r31] Ho SC, Jacob SA and Tangiisuran B (2017) Barriers and facilitators of adherence to antidepressants among outpatients with major depressive disorder: A qualitative study. PLoS One 12(6), 1–19. 10.1371/journal.pone.0179290 PMC5470687 28614368

[r32] Ministry of Health Malaysia. (2017). *National Health and Morbidity Survey 2017: Adolescent Mental Health (DASS-21).*

[r33] Jimmy B and Jose J (2011) Patient medication adherence: Measures in daily practice. Oman Medical Journal 26(3), 155–159. 10.5001/omj.2011.38 22043406 PMC3191684

[r34] Kalaman CR, Ibrahim N, Shaker V, Cham CQ, Ho MC, Visvalingam U, Shahsbuddin FA, Rahman FNA, Halim, MRTA, Kaur M, Azhar FL, Yahya AN, Sham R, Siau CS and Lee KW (2023) Parental factors associated with child or adolescent medication adherence: A systematic review. Healthcare 11(4), 1–32. 10.3390/healthcare11040501 PMC9957533 36833035

[r36] Kvarnström K, Westerholm A, Airaksinen M and Liira H (2021) Factors contributing to medication adherence in patients with a chronic condition: A scoping review of qualitative research. Pharmaceutics 13(7), 1–41. 10.3390/pharmaceutics13071100 PMC8309154 34371791

[r37] Landis JR and Koch GG (1977) The measurement of observer agreement for categorical data. Biometrics 33(1), 159–174. 843571

[r38] Levin JB, Krivenko A, Howland M, Schlachet R and Sajatovic M (2016) Medication adherence in patients with bipolar disorder: A comprehensive review. CNS Drugs 30, 819–835. 10.1007/s40263-016-0368-x 27435356

[r39] Ministry of Health Malaysia. (2014). *Clinical Practice Guidelines: Management of Bipolar Disorder in Adults* (2nd Edn.).

[r40] Ministry of Health Malaysia. (2019). *Clinical Practice Guidelines: Management of Major Depressive Disorder* (2nd Edn.).

[r41] Ministry of Health Malaysia. (2021). *Clinical Practice Guidelines: Management of Schizophrenia* (2nd Edn.).

[r42] Malik M, Kumari, S and Manalai P (2020) Treatment nonadherence: An epidemic hidden in plain sight. Psychiatric Times 37(3), 25–26.

[r43] Marrero RJ, Fumero A, de Miguel A and Penate W (2020) Psychological factors involved in psychopharmacological medication adherence in mentalhealth patients: A systematic review. Patient Education and Counseling 103(10), 2116–2131. 10.1016/j.pec.2020.04.030 32402489

[r44] Mason M (2010) Sample size and saturation in PhD studies using qualitative interviews. Forum Qualitative Sozialforschung Forum: Qualitative Social Research 11(3), 1–19. 10.17169/fqs-11.3.1428

[r45] McMillan SS, Wilson B, Stapleton H and Wheeler AJ (2020) Young people’s experiences with mental health medication: A narrative review of the qualitative literature. Journal of Mental Health 31(2), 281–295. 10.1080/09638237.2020.1714000 32031034

[r46] Morisky DE, Green LW and Levine DM (1986) Concurrent and predictive validity of a self-reported measure of medication adherence. Medical Care 24(1), 67–74. 10.1097/00005650-198601000-00007. 3945130

[r47] Murphy A, Gardner D, Kisely S, Cooke C, Kutcher S and Hughes J (2015) A qualitative study of antipsychotic medication experiences of youth. Journal of the Canadian Academy of Child and Adolescent Psychiatry 24(1), 61–69. 10.1155/2013/390130 26336383 PMC4357337

[r48] Nagae M, Nakane H, Honda S, Ozawa H and Hanada H (2015) Factors affecting medication adherence in children receiving outpatient pharmacotherapy and parental adherence. Journal of Child and Adolescent Psychiatric Nursing 28(2), 109–117. 10.1111/jcap.12113 25989262 PMC6088225

[r49] Neiman AB, Ruppar R, Ho M, Garber L, Weidle PJ, Hong Y, George MG and Thorpe PG (2017) CDC grand rounds: Improving medication adherence for chronic disease management—innovations and opportunities. *Morbidity and Mortality Weekly Report.* https://www.cdc.gov/mmwr/volumes/66/wr/mm6645a2.htm#contribAff 10.15585/mmwr.mm6645a2 PMC5726246 29145353

[r50] Novick D, Montgomery W, Treuer T, Aguado J, Kraemer S and Haro JM (2015) Relationship of insight with medication adherence and the impact on outcomes in patients with schizophrenia and bipolar disorder: Results from a 1-year European outpatient observational study. BMC psychiatry 15(1), 1–8. 26239486 10.1186/s12888-015-0560-4 PMC4524170

[r51] Osterberg L and Blaschke T (2005) Adherence to medication. New England Journal of Medicine 353(5), 487–497. 10.1056/NEJMra050100 16079372

[r52] Phan SV (2016) Medication adherence in patients with schizophrenia. The International Journal of Psychiatry in Medicine 51(2), 211–219. 10.1177/0091217416636601 27079779

[r53] Ramamurthy P, Jayasree A, Solomon S, Rudravaram VV, Menon V and Thilakan P (2023) Medication nonadherence and its associated factors in psychiatric patients in India: A systematic review and meta-analysis. Indian Journal of Psychiatry 65(5), 506–525. 10.4103/indianjpsychiatry.indianjpsychiatry_249_22 37397842 PMC10309262

[r54] Robards F, Kang M, Usherwood T and Sanci L (2018) How marginalized young people access, engage with, and navigate health-care systems in the digital age: Systematic review. Journal of Adolescent Health 62(4), 365–381. 10.1016/j.jadohealth.2017.10.018 29429819

[r55] Semahegn A, Torpey K, Manu A, Assefa N, Tesfaye G and Ankomah A (2020) Psychotropic medication non-adherence and its associated factors among patients with major psychiatric disorders: A systematic review and meta-analysis. Systematic Reviews 9(1), 1–18. 10.1186/s13643-020-1274-3 31948489 PMC6966860

[r56] Stentzel U, van den Berg N, Schulze LN, Schwaneberg T, Radicke F, Langosch JM, Freyberger HJ, Hoffmann W and Grabe H (2018) Predictors of medication adherence among patients with severe psychiatric disorders: Findings from the baseline assessment of a randomized controlled trial (Tecla). BMC Psychiatry 18(1), 1–8. 10.1186/s12888-018-1737-4 29843676 PMC5975380

[r57] Tessier A, Boyer L, Husky M, Bayle F, Llorca PM and Misdrahi D (2017) Medication adherence in schizophrenia: The role of insight, therapeutic alliance and perceived trauma associated with psychiatric care. Psychiatry Research 257, 315–321. 10.1016/j.psychres.2017.07.063 28800510

[r58] Thompson K, Kulkarni J and Sergejew AA (2000) Reliability and validity of a new medication adherence rating scale (MARS) for the psychoses. Schizophrenia Research 42(3), 241–247. 10.1016/s0920-9964(99)00130-9 10785582

[r59] Timlin U, Hakko H, Heino R and Kyngäs H (2015) Factors that affect adolescent adherence to mental health and psychiatric treatment: A systematic integrative review of the literature. Scandinavian Journal of Child and Adolescent Psychiatry and Psychology 3, 99–107. 10.21307/sjcapp-2015-010

[r60] Tong A, Sainsbury P and Craig J (2007) Consolidated criteria for reporting qualitative research (COREQ): A 32-item checklist for interviews and focus groups. International Journal for Quality in Health Care 19(6), 349–357. 10.1093/intqhc/mzm042 17872937

[r61] Wang H, Yao F, Wang H, Wang C and Guo Z (2022) Medication adherence and influencing factors among patients with severe mental disorders in low-income families during COVID-19 outbreak. Frontiers in Psychiatry 12, 1–9. 10.3389/fpsyt.2021.799270 PMC8803649 35115971

[r62] World Health Organization (WHO) (2003) Adherence to Long Term Therapies: Evidence for Action. Geneva: WHO.

[r63] World Health Organization (2021) *Mental Disorders.* Available at https://www.who.int/news-room/fact-sheets/detail/mental-disorders (accessed 12 December 2023).

[r64] Zarea K, Fereidooni-Moghadam M and Hakim A (2016) Adherence to medication regimen in patients with severe and chronic psychiatric disorders: A qualitative study. Issues in Mental Health Nursing 37(11), 868–874. 10.1080/01612840.2016.1239147 27898289

