# Supplementary material for: Challenges and strategies for improving medication adherence among adolescent psychiatric patients: A qualitative study
Source: Glob Ment Health (Camb). 2024 Dec 2;11:e117. doi: 10.1017/gmh.2024.80 (PMC11704366; doi:10.1017/gmh.2024.80)
Supplement: Peer review history

# Editor report: Recommendation: Challenges and strategies for improving medication adherence among adolescent psychiatric patients: A qualitative study — R0/PR2

**DOI:** 10.1017/gmh.2024.80.pr2

**Affiliations:** Director, National Institute of Mental Health, Czech Republic

No accompanying comment.

# Editor report: Decision: Challenges and strategies for improving medication adherence among adolescent psychiatric patients: A qualitative study — R0/PR3

**DOI:** 10.1017/gmh.2024.80.pr3

**Affiliations:** London School of Hygiene & Tropical Medicine, United Kingdom of Great Britain and Northern Ireland

No accompanying comment.

# Editor report: Recommendation: Challenges and strategies for improving medication adherence among adolescent psychiatric patients: A qualitative study — R1/PR5

**DOI:** 10.1017/gmh.2024.80.pr5

**Affiliations:** Director, National Institute of Mental Health, Czech Republic

Thank you for revising the paper. We appreciate your efforts. Regarding publication, we have noticed some conflicting statements in the discussion and conclusion sections that need clarification before proceeding.

The discussion section states: ‘In contrast to WHO’s multifactorial phenomenon that highlights socioeconomic-related factors towards adherence, the present qualitative study did not identify any connection between factors pertaining to adolescents’ demographic or socioeconomic profile and medication adherence (WHO, 2003).’

However, the conclusion section concludes: ‘In summary, the outcomes of this qualitative study (e.g., social factors, health care team/system factors, therapy-related factors, and patient-related factors) align with the World Health Organization’s multifactorial approach to adherence.’

Could you please provide clarification on these conflicting statements? Once resolved, we will proceed with the publication process. Thank you.

# Editor report: Decision: Challenges and strategies for improving medication adherence among adolescent psychiatric patients: A qualitative study — R1/PR6

**DOI:** 10.1017/gmh.2024.80.pr6

**Affiliations:** London School of Hygiene & Tropical Medicine, United Kingdom of Great Britain and Northern Ireland

No accompanying comment.

# Editor report: Recommendation: Challenges and strategies for improving medication adherence among adolescent psychiatric patients: A qualitative study — R2/PR8

**DOI:** 10.1017/gmh.2024.80.pr8

**Affiliations:** Director, National Institute of Mental Health, Czech Republic

No accompanying comment.

# Editor report: Decision: Challenges and strategies for improving medication adherence among adolescent psychiatric patients: A qualitative study — R2/PR9

**DOI:** 10.1017/gmh.2024.80.pr9

**Affiliations:** London School of Hygiene & Tropical Medicine, United Kingdom of Great Britain and Northern Ireland

No accompanying comment.
